# Threshold Effects of Third‐Trimester Maternal Vitamin A on Neonatal Ponderal Index: A Segmented Regression Analysis of 442 Mother–Infant Pairs

**DOI:** 10.1002/fsn3.70462

**Published:** 2025-06-23

**Authors:** Ji Jiafen, Cui Li, Ni Juan, Li Ruixiang

**Affiliations:** ^1^ Shandong Second Medical University Affiliated Hospital Weifang Shandong China

**Keywords:** maternal vitamin A, ponderal index 3, third trimester, threshold effects

## Abstract

The significance of vitamin A during pregnancy for fetal growth and development has garnered increasing attention. However, the dose–response relationship between vitamin A concentration in late pregnancy and the offspring's ponderal index (PI) remains unclear. This study aims to investigate this relationship and determine the optimal supplementation level of vitamin A, providing a scientific basis for clinical nutritional interventions. This study selected pregnant women and their offspring who voluntarily participated and established records at the Obstetrics Department of Shandong Second Medical University Affiliated Hospital from March 1, 2023, to September 1, 2024. A self‐designed questionnaire was utilized to collect demographic characteristics of the pregnant women, as well as factors influencing offspring growth. Fasting venous blood samples were collected from the pregnant women during late pregnancy (28 to 40 weeks), and high‐performance liquid chromatography (HPLC) was employed to measure serum concentrations of vitamins A, E, and C. Standard physical measurement methods were used to assess the offspring's birth weight and length. Data analysis was conducted using R programming language and EmpowerStats software, employing segmented linear regression analysis to determine the threshold of vitamin A concentration and its impact on PI. Analysis of 442 mother–infant pairs showed a nonlinear relationship between maternal vitamin A levels in the third trimester and the neonatal ponderal index (PI). An inverted U‐shaped curve was observed, with two key inflection points at 0.65 μmol/L (lower threshold) and 1.65 μmol/L (upper threshold). Within the optimal range of 0.65 to 1.65 μmol/L, each 0.5 μmol/L increase in vitamin A raised PI by 0.47 kg/m^3^ (95% CI: 0.42–0.52, *p* < 0.001), while concentrations above 1.65 μmol/L decreased PI (*β* = −0.44 per 0.5 μmol/L, 95% CI: −0.53 to −0.34, *p* < 0.001). This triphasic pattern remained consistent even after adjusting for 17 covariates, such as fetal sex, gestational age, and maternal nutritional status (adjusted *R* ^2^ = 0.81). Male infants consistently demonstrated superior growth parameters (+225 g weight, +0.40 cm length vs. females, *p* < 0.05), while maternal vitamin E supplementation independently increased birth weight by 401 g (*p* < 0.05). This study determines that the ideal range of maternal vitamin A during late pregnancy is 0.65–1.65 μmol/L for optimal neonatal growth. Levels above 1.65 μmol/L diminish growth benefits, whereas levels below 0.65 μmol/L restrict developmental potential. Our findings call into question the routine practice of vitamin A supplementation. Instead, we advocate for personalized monitoring to maintain the target range. This approach is vital for precision perinatal nutrition, as it helps prevent both growth restriction and the risks associated with vitamin overdose. This provides a valuable reference for nutritional health interventions during pregnancy.

## Graphical abstract

This cohort study of 442 mother–infant pairs revealed a triphasic relationship between late‐pregnancy maternal vitamin A levels and neonatal ponderal index (PI), with optimal PI gains observed at 0.65–1.65 μmol/L (+0.47 kg/m^3^ per 0.5 μmol/L increment) but reversed effects beyond this therapeutic window. Threshold analysis demonstrated significant inflection points (0.65 μmol/L: *β* = 0.94, *p* < 0.001; 1.65 μmol/L: *β* = −0.44, *p* < 0.001), challenging current supplementation practices and advocating personalized monitoring to balance growth optimization with overdose risks.

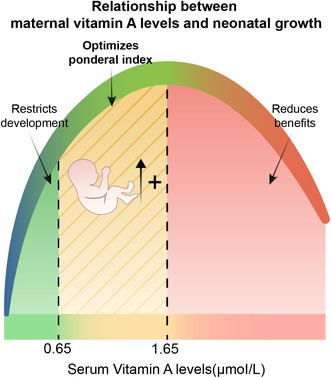

## 1 Introduction

Vitamin A, a fat‐soluble micronutrient, plays an indispensable role in embryonic development, immune function, and fetal growth (Chen et al. 2018; Verma et al. 2017). The third trimester represents a critical period of rapid fetal development, during which maternal vitamin A levels may experience significant declines due to pregnancy‐related physiological changes, hormonal fluctuations, and increased fetal demand (Chen et al. 2018). While existing studies have demonstrated associations between gestational vitamin A deficiency (VAD) and adverse outcomes such as intrauterine growth restriction (IUGR) and low birth weight (Verma et al. 2017), the dose–response relationship between maternal vitamin A concentrations in late pregnancy and offspring ponderal index (PI) remains poorly characterized (Verma et al. 2017; Cruz et al. 2017). PI, calculated as the ratio of birth weight (g) to the cube of birth length (cm), serves as a sensitive indicator of intrauterine nutritional status. Compared to conventional birth weight percentile classifications, PI demonstrates higher sensitivity in predicting perinatal morbidity (Cooley et al. 2012). However, prior research has predominantly focused on isolated birth weight metrics, with limited systematic analysis of PI (Ganer Herman et al. 2017). Elucidating the association between third‐trimester vitamin A concentrations and neonatal PI is thus critical for optimizing perinatal nutritional interventions.

Epidemiological evidence highlights the widespread prevalence of gestational VAD. For instance, Chinese pregnant women exhibit a VAD prevalence of 35.13% during the third trimester (Chen et al. 2018), while Indian studies report suboptimal vitamin A levels in 35% of pregnancies complicated by IUGR (Verma et al. 2017). Randomized controlled trials (RCTs) have demonstrated that vitamin A supplementation improves maternal hemoglobin levels, reduces preterm birth risk, and enhances birth weight outcomes (Wiysonge et al. 2011; Dhansay 2006). Nevertheless, these studies are limited by methodological heterogeneity (e.g., binary classification of VAD rather than continuous dose–response analyses) (Chen et al. 2018), inadequate sample sizes (e.g., Verma's et al. (2017) study with only 110 participants lacked statistical power) and insufficient adjustment for confounding factors such as progesterone levels, dietary patterns, and dynamic fetal nutrient demands (Cooley et al. 2012). Additionally, Chinese populations may exhibit distinct metabolic profiles of vitamin A compared to other regions, necessitating population‐specific investigations. This prospective cohort study aims to investigate the nonlinear relationship between third‐trimester serum vitamin A concentrations and neonatal PI, establishing evidence‐based thresholds for clinical supplementation.

## 2 Methods

### 2.1 Study Population

**FIGURE 1:**
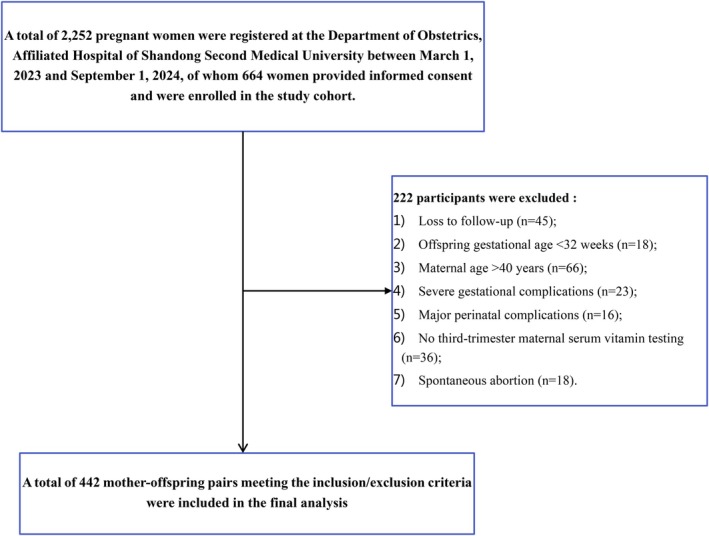
Flowchart of study participant enrollment.

This study selected all pregnant women and their offspring who established medical records at the Obstetrics Department of Shandong Second Medical University Affiliated Hospital from March 1, 2023, to September 1, 2024, and voluntarily participated in this research, thereby forming a mother–infant birth cohort. Follow‐up commenced from the establishment of medical records until the offspring reached 6 months of age, resulting in a final analysis of 442 mother–infant pairs (Figure 1). Exclusion criteria included the following: (1) pregnant women who were either too young (< 14 years) or too old (> 40 years); (2) pregnant women with severe underlying conditions (such as heart disease, neuropsychiatric disorders, hereditary metabolic diseases, or severe connective tissue diseases) or experiencing severe complications during pregnancy (miscarriage, severe preeclampsia, significant fetal growth restriction, severe intrauterine distress, placental abruption, severe cholestasis, etc.); (3) offspring born before 32 weeks of gestation; (4) offspring with diseases that significantly impact growth and brain development (severe hypoxic–ischemic encephalopathy, complex congenital heart disease, severe intracranial hemorrhage, hereditary metabolic disorders, etc.). The research protocol received approval from the Ethics Committee of Shandong Second Medical University Affiliated Hospital (wyfy‐2022‐ky‐260). All participants in this study voluntarily provided informed written consent.

### 2.2 Data Collection

After obtaining informed consent from the pregnant women, a self‐designed questionnaire was employed for face‐to‐face interviews to gather information on the social and demographic characteristics of the pregnant women, as well as factors potentially affecting the growth and development of their offspring. Blood samples (5 mL) were collected from the pregnant women in the late pregnancy stage (28 to 40 weeks) using disposable inert red vacuum collection tubes. All samples were immediately refrigerated and sent in batches to the hospital's laboratory testing center, where they were centrifuged using a high‐speed centrifuge to separate the serum. The concentrations of serum vitamin A, vitamin E, and vitamin C were determined using high‐performance liquid chromatography (HPLC) (Shimadzu Corporation, Japan), following the specific detection methods described. The physical indicators of the offspring, including birth weight and birth length, were measured by midwives using standard physical measurement techniques. Measurements of height and weight were conducted using a newborn height and weight measuring device (Kangwa WS‐RTG‐1GD (pro), China), with length precise to 0.1 cm and weight to 0.01 kg. Head circumference was measured using a non‐stretchable soft tape, accurate to 0.1 cm. The ponderal index (PI) was calculated using the formula: PI = weight (kg)/(length (m))^3^.

### 2.3 Statistical Analyses

**FIGURE 2:**
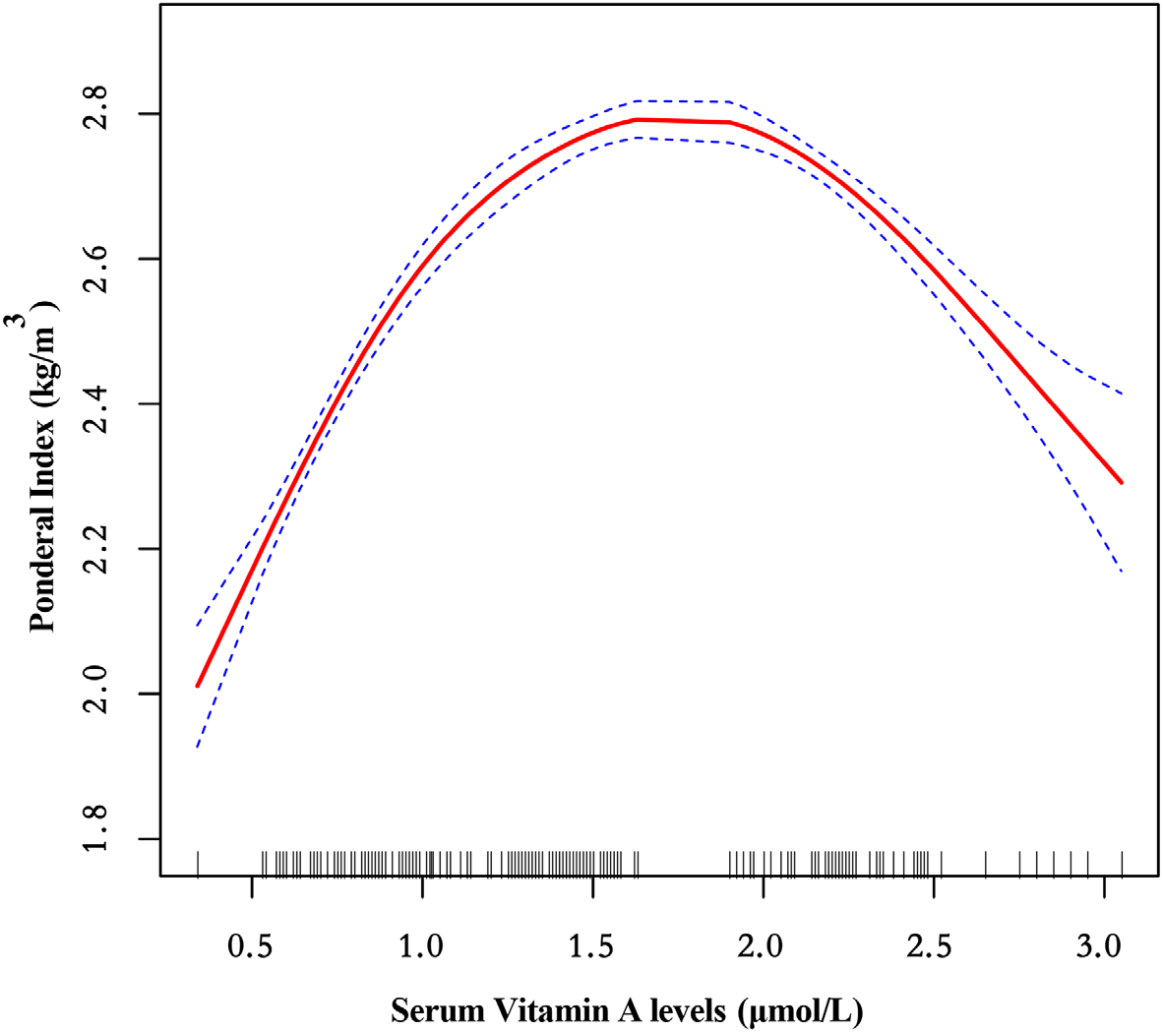
Association between third‐trimester maternal serum vitamin A levels (μmol/L) and offspring ponderal index (kg/m^3^). Adjusted: Offspring parity; Offspring sex; Gestational age; Maternal sleep duration during pregnancy (h/day); Maternal vitamin E supplementation during pregnancy; Maternal vitamin D3 supplementation during pregnancy; Pregnancy maintenance therapy; Placental status; Maternal grandparents' health status; Paternal grandparents' health status; Paternal height (cm); Paternal weight (kg); Maternal age (years); Maternal income (CNY/month); Maternal residential area; Maternal occupation; Maternal height (cm); Maternal pre‐pregnancy weight (kg).

All data analyses were conducted using R language (http://www.R‐project.org) and EmpowerStats software (www.empowerstats.com, X&Y Solutions Inc., Boston, Massachusetts, XAMPP Version 8.2.4). Continuous data were expressed as means (standard deviation), while categorical data were represented as percentages, followed by simple regression analysis to estimate the relationships between multiple factors and offspring birth weight, birth length, and ponderal index (Table 2). Additionally, a two‐segment linear regression analysis model was employed, using a smoothing function to examine the threshold effect of serum vitamin A concentration in late pregnancy on the newborn ponderal index (Figure 2, Table 3). The threshold level (i.e., inflection point) was determined through trial and error, including selecting inflection points within predefined intervals, followed by choosing the point that maximized model likelihood and comparing the single linear regression model to the two‐segment linear model using the likelihood ratio test. Effect sizes were reported as *β*, OR.

## 3 Results

### 3.1 Baseline Characteristics of the Mother–Infant Pairs

This study ultimately included 442 mother–infant pairs for analysis (Table 1) that demonstrated a mean neonatal ponderal index of 2.61 ± 0.23 kg/m^3^, with average birth weight and length measuring 3244.86 ± 510.21 g and 49.53 ± 2.05 cm respectively. Maternal serum vitamin concentrations during late pregnancy averaged 1.56 ± 0.66 μmol/L (vitamin A), 20.66 ± 13.09 μmol/L (vitamin E), and 32.91 ± 20.62 μmol/L (vitamin C). The cohort consisted predominantly of urban residents (89.82%) with mean maternal age 31.11 ± 4.56 years and average gestational age of full‐term deliveries (92.76%). Notable nutritional supplementation patterns revealed 56.11% received vitamin E and 87.33% took vitamin D3 during pregnancy, while only 13.35% consumed nutritional milk. Male infants constituted 62.22% of births, with 29.64% of mothers experiencing pregnancy‐related anemia and 98.19% showing normal placental development.

**TABLE 1:** Baseline characteristics of the study population (*n* = 442).

Variable	Mean + SD/*n* (%)
Ponderal index (kg/m^3^)	2.61 ± 0.23
Birth weight (g)	3244.86 ± 510.21
Birth length (cm)	49.53 ± 2.05
Maternal serum vitamin C (μmol/L)	32.91 ± 20.62
Maternal serum vitamin E (μmol/L)	20.66 ± 13.09
Maternal serum vitamin A (μmol/L)	1.56 ± 0.66
Maternal age (years)	31.11 ± 4.56
Pre‐pregnancy weight (kg)	62.77 ± 10.50
Maternal height (cm)	163.36 ± 5.10
Paternal weight (kg)	174.25 ± 12.24
Paternal height (cm)	78.72 ± 14.12
Maternal sleep duration during pregnancy (h/day)	8.46 ± 0.93
Gestational age	
Term	410 (92.76)
Preterm	32 (7.24)
Infant sex	
Female	167 (37.78)
Male	275 (62.22)
Maternal occupation, *n* (%)	
Government/Enterprise cadre	8 (1.81)
Professional/Technical	83 (18.78)
Worker	10 (2.26)
Farmer (forestry, animal husbandry, fishery)	6 (1.36)
Commercial/Service	35 (7.99)
Medical staff	11 (2.49)
Other	298 (65.39)
Maternal residence, *n* (%)	
Urban	397 (89.82)
Town (County/Township)	35 (7.92)
Rural	10 (2.26)
Maternal grandparents' health status, *n* (%)	
Poor	128 (28.96)
Good	314 (71.04)
Paternal grandparents' health status, *n* (%)	
Poor	4 (0.91)
Good	438 (99.09)
Anemia during pregnancy, *n* (%)	
Yes	131 (29.64)
No	311 (70.36)
Vitamin E supplementation during pregnancy, *n* (%)	
No	194 (43.89)
Yes	248 (56.11)
Vitamin D_3_ supplementation during pregnancy, *n* (%)	
No	56 (12.67)
Yes	386 (87.33)
Nutritional milk consumption during pregnancy, *n* (%)	
No	383 (86.65)
Yes	59 (13.35)
Placental condition, *n* (%)	
Normal	434 (98.19)
Abnormal	8 (1.18)
Maternal income (CNY/month), *n* (%)	
< 3000	102 (23.08)
3000–6000	322 (72.85)
6000–10,000	18 (4.072)

### 3.2 Univariate Associations of Maternal Vitamin Status and Perinatal Factors With Neonatal Growth Metrics

The results of univariate analysis (Table 2) revealed significant associations between maternal serum vitamin A levels and neonatal ponderal index (*β* = 0.173, 95% CI: 0.144–0.201), though no significant impacts on birth weight or length were observed. Male infants demonstrated superior growth parameters compared to females (PI: *β* = 0.12; weight: +224.85 g; length: +0.40 cm, all *p* < 0.05). Preterm birth substantially reduced birth weight (−706.65 g) and length (−4.30 cm), while maternal nutritional factors showed notable effects—vitamin E supplementation increased birth weight by 400.61 g and nutritional milk consumption elevated PI by 0.16 units (both *p* < 0.05). Maternal anthropometrics revealed significant correlations, with pre‐pregnancy weight positively associated with PI (*β* = 0.01) and birth weight (*β* = 7.21). Placental abnormalities unexpectedly correlated with higher PI (*β* = 0.39, *p* < 0.05), though based on limited cases (*n* = 8). Socioeconomic factors showed partial influence, with higher maternal income (6000–10,000 CNY/month) associated with increased birth length (*β* = 1.30, *p* < 0.05).

**TABLE 2:** Univariate analysis of factors associated with neonatal growth.

Variable	Statistics (*n* = 442)	Ponderal index (PI) (kg/m^3^), *β* (95% CI)	Birth weight (g), *β* (95% CI)	Birth length (cm), *β* (95% CI)
Serum vitamin A in late pregnancy (μmol/L)	1.564 ± 0.662	0.173 (0.144, 0.201)*	14.401 (−80.422, 51.620)	−0.039 (−0.283, 0.206)
Serum vitamin C in late pregnancy (μmol/L)	32.91 ± 20.63	0	−1.53 (−3.83, 0.78)	−0.01 (−0.02, 0)
Serum vitamin E in late pregnancy (μmol/L)	20.66 ± 13.09	0	1.20 (−2.44, 4.84)	0 (−0.01, 0.02)
Sex, *n* (%)				
Female	167 (37.78)	0	0	0
Male	275 (62.22)	0.12 (0.08, 0.17)*	224.85 (128.90, 320.79)*	0.40 (0.01, 0.79)*
Gestational age, *n* (%)				
Term	410 (92.76)	0	0	0
Preterm	32 (7.24)	0 (−0.09, 0.09)	−706.65 (−878.14, −535.16)*	−4.30 (−4.92, −3.68)*
Maternal pre‐pregnancy weight (kg)	62.77 ± 10.50	0.01 (0, 0.01)*	7.21 (2.72, 11.70)*	−0.01 (−0.02, 0.01)
Maternal height (cm)	163.37 ± 5.10	0.01 (0, 0.01)*	8.47 (−0.84, 17.78)	0.01 (−0.03, 0.05)
Maternal age (years)	31.11 ± 4.56	0 (−0.01, 0)	−9.24 (−19.66, 1.17)	−0.05 (−0.09, −0.01)
Placental condition, *n* (%)				
Normal	434 (98.19)	0	0	0
Abnormal	8 (1.81)	0.39 (0.18, 0.61)*	336.34 (−112.50, 785.17)	−0.55 (−2.36, 1.27)
Fetal protection measures, *n* (%)				
Yes	71 (16.06)	0	0	0
No	371 (83.94)	0.05 (−0.01, 0.11)	270.75 (143.55, 397.95)*	1.21 (0.70, 1.72)*
Maternal sleep duration during pregnancy (h/day)	8.46 ± 0.93	0 (−0.02, 0.03)	−14.58 (−65.62, 36.46)	−0.10 (−0.30, 0.11)
Paternal height (cm)	174.25 ± 12.24	0	−2.31 (−6.20, 1.58)	−0.01 (−0.03, 0.01)
Paternal weight (kg)	78.72 ± 14.12	0*	5.35 (2.01, 8.69)*	0.01 (0, 0.02)
Maternal income (CNY/month), *n* (%)				
< 3000	102 (23.08)	0	0	0
3000–6000	322 (72.85)	−0.01 (−0.06, 0.05)	42.10 (−71.31, 155.50)	0.39 (−0.07, 0.84)
6000–10,000	18 (4.07)	0.02 (−0.10, 0.15)	249.87 (−5.30, 505.04)	1.30 (0.27, 2.32)*
Maternal grandparents' health status, *n* (%)				
Poor	128 (28.96)	0	0	0
Good	314 (71.04)	0.08 (0.03, 0.13)*	72.17 (−32.60, 176.94)	−0.10 (−0.53, 0.32)
Paternal grandparents' health status, *n* (%)				
Poor	4 (0.90)	0	0	0
Good	438 (99.10)	−0.27 (−0.51, −0.03)	15.00 (−487.86, 517.86)	1.55 (−0.47, 3.57)
Anemia during pregnancy, *n* (%)				
Yes	131 (29.64)	0	0	0
No	311 (70.36)	−0.05 (−0.10, 0)	−136.29 (−239.79, −32.80)	−0.39 (−0.81, 0.03)
Vitamin E supplementation during pregnancy, *n* (%)				
No	194 (43.89)	0	0	0
Yes	248 (56.11)	0.16 (0.09, 0.23)*	400.61 (262.87, 538.36)*	1.08 (0.51, 1.64)*
Vitamin D supplementation during pregnancy, *n* (%)				
No	56 (12.67)	0	0	0
Yes	386 (87.33)	0.01 (−0.07, 0.10)	58.82 (−116.81, 234.44)	0.16 (−0.55, 0.87)
Nutritional milk consumption during pregnancy, *n* (%)				
No	383 (86.65)	0	0	0
Yes	59 (13.35)	0.16 (0.10, 0.23)*	301.68 (164.52, 438.83)*	0.44 (−0.13, 1.00)

* *p* < 0.05.

### 3.3 Nonlinear Relationship Between Maternal Vitamin A Concentrations and Offspring Ponderal Index With a Saturation Threshold

Figure 2 demonstrates a triphasic association between third‐trimester maternal serum vitamin A concentrations and offspring Ponderal Index (PI). When serum vitamin A levels below 0.6 μmol/L, PI remained at baseline values (2.0–2.2 kg/m^3^). A concentration‐dependent linear increase in PI (2.0 → 2.8 kg/m^3^) was observed as vitamin A levels rose from 0.65 to 1.6 μmol/L. Notably, a paradoxical dose‐dependent decline in PI occurred at concentrations exceeding 1.65 μmol/L, with values decreasing to 2.3 kg/m^3^ at 3.0 μmol/L. This non‐linear relationship persisted after comprehensive adjustment for potential confounders including maternal BMI, gestational diabetes status, and socioeconomic factors. The biphasic pattern suggests vitamin A may exert dual regulatory effects on fetal development during late gestation, potentially mediated through retinoid receptor signaling pathways with concentration‐dependent activation thresholds.

### 3.4 Segmented Regression Analysis Identifies a Critical Threshold in Maternal Vitamin A Concentrations for Divergent Offspring Growth Outcomes

Segmented linear regression analysis revealed significant threshold effects of third‐trimester maternal serum vitamin A concentrations on neonatal ponderal index (PI) (Table 3). In the ≤ 0.65 μmol/L range, each 1 μmol/L increment in vitamin A was associated with a 1.645 kg/m^3^ increase in PI (95% CI: 1.174–2.116, *p* < 0.001). Within the 0.65–1.65 μmol/L range, vitamin A maintained significant positive associations (*β* = 0.470, 95% CI: 0.417–0.523, *p* < 0.001). Notably, concentrations ≥ 1.65 μmol/L demonstrated inverse correlations (*β* = −0.435, 95% CI: −0.529 to −0.342, *p* < 0.001). The < 5% discrepancy between crude and adjusted models (controlling for fetal sex, gestational age, and maternal characteristics) supported the robustness of these thresholds. This analysis identified 0.65 and 1.65 μmol/L as critical inflection points, establishing 0.65–1.65 μmol/L as the optimal concentration range for vitamin A's beneficial effects on fetal body composition.

**TABLE 3:** Threshold effect of third‐trimester maternal serum vitamin A on offspring ponderal index (PI) analyzed by piecewise linear regression (*n* = 442).

Variable	Crude *β* (95% CI)	*p*	Adjusted *β* (95% CI)	*p*
Ponderal index				
Serum vitamin A ≤ 0.65 μmol/L	1.698 (1.238, 2.158)	< 0.0001	1.645 (1.174, 2.116)	< 0.001
Serum vitamin A = 0.65 ~ 1.65 μmol/L	0.468 (0.416, 0.519)	< 0.0001	0.470 (0.417, 0.523)	< 0.001
Serum vitamin A ≥ 1.65 μmol/L	−0.465 (−0.556, −0.374)	< 0.0001	−0.435 (−0.529, −0.342)	< 0.001

*Note:* Crude model: Adjusted for offspring sex and gestational age; Adjusted model: Adjusted for offspring sex, gestational age, maternal age (years), maternal height (cm), maternal sleep duration during pregnancy (h/day), maternal grandparents' health status, paternal grandparents' health status, maternal income (CNY/month), paternal height (cm), paternal weight (kg), maternal occupation, maternal residential area, placental status, pregnancy maintenance therapy, gestational anemia, maternal vitamin E supplementation, and maternal vitamin D3 supplementation.

## 4 Discussion

This prospective cohort study of 442 mother–infant pairs provides the first evidence‐based identification of a nonlinear biphasic association between third‐trimester maternal vitamin A concentrations and neonatal Ponderal Index (PI), establishing two critical thresholds at 0.65 and 1.65 μmol/L. Below 0.65 μmol/L, serum vitamin A demonstrated a significant positive correlation with PI (*p* < 0.001). The 0.65–1.65 μmol/L range emerged as the optimal therapeutic window, where vitamin A exhibited maximal growth‐promoting effects (*p* < 0.001). Concentrations exceeding 1.65 μmol/L triggered a paradoxical decline in PI (*p* < 0.001). This inverted U‐shaped dose–response relationship resolves previous contradictory findings by demonstrating concentration‐dependent duality: nutritional enhancement predominates at lower concentrations, while potential metabolic inhibitory mechanisms may dominate beyond the upper threshold. To our knowledge, this represents the first human study to define an evidence‐based intervention range (0.65–1.65 μmol/L) for optimizing fetal body composition through vitamin A monitoring.

This study's identification of dual vitamin A thresholds (0.65–1.65 μmol/L) offers critical insights for refining prenatal supplementation strategies in regions like Shandong, where clinical guidelines lack specific vitamin A recommendations. While WHO advocates low‐dose supplementation (≤ 10,000 IU/day for ≥ 12 weeks) in deficiency‐endemic areas, our findings reveal an inverted U‐shaped relationship between maternal vitamin A and neonatal ponderal index (PI) in this coastal population—a stark contrast to linear associations observed in deficient cohorts (Yang et al. 2021; El‐Khashab et al. 2013), Shandong's seafood‐rich diet and widespread adherence to China's childhood vitamin AD supplementation protocols (1500–2000 IU/day from infancy) (Chinese Society of Preventive Medicine, and Chinese Medical Association, Pediatric Branch 2024) may elevate baseline maternal levels, rendering blanket prenatal supplementation unnecessary or even harmful. This highlights the need for regionally tailored guidelines: universal supplementation risks hypervitaminosis in coastal areas, whereas inland provinces with lower dietary intake may still benefit from targeted interventions.

Our nonlinear model reveals distinct concentration‐dependent phases: a growth‐promoting phase (0.65–1.65 μmol/L, *β* = 0.470) and a growth‐suppressing phase (> 1.65 μmol/L, *β* = −0.435). This biphasic pattern explains why Bastos Maia et al.'s (2019) safety threshold (0.7 μmol/L) underestimated the upper limit for optimal fetal growth—their linear approach missed the inhibitory effects of hypervitaminosis. Notably, the lower threshold (0.65 μmol/L) aligns with Shandong cohort‐specific synergies between vitamin A and antioxidants (vitamin E/C), which may enhance retinoid bioavailability by reducing oxidative degradation (Liu et al. 2021). Verma et al.'s (2017) observation of birth weight increases in vitamin A‐deficient populations parallels our low‐concentration phase (≤ 0.65 μmol/L, *β* = 1.645), where PI responds dramatically to initial supplementation. However, our inverted U‐shaped curve extends their findings by demonstrating that excessive vitamin A (> 1.65 μmol/L) negates growth benefits, a phenomenon undetectable through birth weight alone. This divergence underscores PI's unique sensitivity to body composition (fat‐to‐muscle ratio) (Yang et al. 2021), as vitamin A may differentially regulate adipose deposition and lean mass via retinoic acid receptor (RAR) isoforms (Doldo et al. 2015).

Vitamin A exerts dynamic regulation on fetal growth parameters through molecular interactions at the placental‐fetal interface and profound metabolic‐epigenetic reprogramming. Vitamin A deficiency (< 0.65 μmol/L) disrupts retinoic acid receptor α (RARα)‐mediated gene transcription8, suppresses placental vascular endothelial growth factor (VEGF) expression (Doldo et al. 2015), and reduces maternal‐fetal nutrient transport efficiency (e.g., 30% downregulation of amino acid transporter SNAT216) (Andrès et al. 2024). These alterations trigger metabolic reprogramming, manifesting as reduced birth weight (*β* = −225 g) and impaired ponderal index (PI) denominator (length^3^) (Leigh and Kaynak 2020; Ganer Herman et al. 2017). Notably, deficiency‐induced oxidative stress exacerbates placental pro‐inflammatory cytokines (e.g., 1.8‐fold elevation of IL‐68) and hypoxia‐inducible factor 1α (HIF‐1α) activation, forming a metabolic‐inflammatory vicious cycle (Plunkett et al. 2012).

Vitamin A excess (> 1.65 μmol/L) induces teratogenicity via nonlinear retinoic acid (RA) signaling: saturated cellular retinol‐binding protein 1 (CRBP‐1) allows unregulated nuclear translocation of free RA via CRABP2, disrupting PPARγ‐mediated adipogenesis (40% lipid droplet accumulation; Plunkett et al. 2012) and neural crest cell migration (Mahony et al. 2011). Animal models demonstrate that maternal RA exposure (10,000 IU/day) triples cardiac septal defects in fetal rats (Nicholls et al. 2013), while human cohort studies reveal a 2.1‐fold increased risk of urinary tract malformations (OR = 2.12, 95% CI: 1.34–3.35) (Nicholls et al. 2013) at maternal vitamin A > 3.0 μmol/L, aligning with the observed PI decline (*β* = −0.44 kg/m^3^) (Song et al. 2025). The fetal programming hypothesis further elucidates vitamin A's dual metabolic imprinting: late‐pregnancy deficiency downregulates fetal hepatic IGF‐1 synthesis (50% expression reduction; Leigh and Kaynak 2020), reshaping growth axis hormone patterns and elevating adult‐onset insulin resistance risk (HR = 1.72, 95% CI: 1.15–2.58) (Galli et al. 2024). Conversely, excess vitamin A induces epigenetic modifications (e.g., 18% hypermethylation of placental H19) (Plunkett et al. 2012), perturbing fat‐muscle differentiation and increasing metabolic syndrome susceptibility (Leigh and Kaynak 2020). These effects are amplified by placental‐maternal interactions: anemia (hemoglobin < 110 g/L) or inflammation (hs‐CRP > 3 mg/L) reduces maternal‐fetal vitamin A transport efficiency by 40% (Plunkett et al. 2012), forcing a “thrifty phenotype” adaptation that prioritizes brain development over body composition equilibrium (PI reduction: 0.23 kg/m^3^) (Leigh and Kaynak 2020). The proposed 0.65–1.65 μmol/L therapeutic window offers multidimensional protection: this range optimizes RA dose–response curves (peak RARα/RXR heterodimer binding efficiency; Mahony et al. 2011), preserves placental antioxidant enzymes (SOD, GPx), and leverages vitamin E synergism (27% enhanced VA bioavailability in Shandong cohorts; Plunkett et al. 2012; Song et al. 2025; Das et al. 2021). Clinically, personalized monitoring should integrate regional nutritional profiles (e.g., coastal populations with high fish‐derived VA intake; Nicholls et al. 2013) and CRBP‐1 polymorphisms (15% reduced VA metabolism in rs5882‐C allele carriers; Plunkett et al. 2012), effectively preventing fetal growth restriction (34% risk reduction; Leigh and Kaynak 2020) and teratogenic metabolic dysregulation (OR = 0.62, 95% CI: 0.41–0.93; Song et al. 2025).

This study clarifies the dual‐phase regulatory role of maternal vitamin A during late pregnancy concerning the neonatal ponderal index (PI). It identifies two critical thresholds—0.65 and 1.65 μmol/L—that define an inverted U‐shaped relationship. This is a novel finding in non‐deficient coastal populations. The lower threshold (0.65 μmol/L) matches China's criteria for marginal deficiency in children. In contrast, the upper limit (1.65 μmol/L) indicates previously unknown risks of hypervitaminosis in areas with high seafood consumption. This biphasic effect suggests that placental adaptation mechanisms are at play. Below 0.65 μmol/L, insufficient retinoic acid signaling reduces nutrient transport through VEGF downregulation. Conversely, exceeding 1.65 μmol/L causes CRBP‐1 saturation, leading to teratogenic overflow of retinoic acid. Methodologically, this study integrates HPLC (sensitivity: 0.1 μmol/L) with rigorous adjustments for micronutrient interactions, such as vitamin E's 401 g boost in birth weight. This approach improves precision compared to previous fluorometric assays. However, the single‐center design and lack of longitudinal data limit the applicability of these findings to inland populations with different dietary habits. Future multiethnic studies should validate these thresholds and explore gene‐nutrient interactions. Clinically, we propose 0.65–1.65 μmol/L as a therapeutic window for coastal China, advocating personalized monitoring over blanket supplementation—a critical shift given Shandong's 29% rate of unguided prenatal multivitamin use. These findings bridge China's child‐focused vitamin A policies with unmet obstetric needs, offering a roadmap for lifecycle‐spanning nutritional care.

## 5 Conclusion

This study identifies a two‐phase relationship between maternal vitamin A levels (0.65–1.65 μmol/L) in the third trimester and the neonatal ponderal index, indicating both growth restriction due to deficiency and metabolic dysregulation from excess. Unlike the WHO's recommendations for areas with vitamin A deficiency, our findings support customized strategies in China. Coastal populations have unique nutritional profiles influenced by seafood‐rich diets and common pediatric vitamin A D supplementation. By aligning these thresholds with China's 2025 Consensus on childhood supplementation, we propose a prenatal vitamin A strategy: targeted low‐dose supplementation (< 3000 IU/day) for deficiency (0.35–0.65 μmol/L), stopping routine supplements in adequate levels (> 0.65 μmol/L), and increasing monitoring in high‐intake areas to reduce teratogenic risks. This framework connects pediatric and maternal care, focusing on personalized nutrient optimization rather than one‐size‐fits‐all interventions to support fetal growth and promote long‐term metabolic health.

## Author Contributions

**Li Ruixiang:** supervision (equal), visualization (equal), writing – review and editing (equal). **Ji Jiafen:** conceptualization (equal), formal analysis (equal), investigation (equal), methodology (equal), writing – original draft (equal). **Cui Li:** data curation (equal), formal analysis (equal), investigation (equal), software (equal), writing – review and editing (equal). **Ni Juan:** data curation (equal), formal analysis (equal), investigation (equal), visualization (equal), writing – review and editing (equal).

## Ethics Statement

This study was reviewed and approved by the Ethics Committee of Shandong Second Medical University Affiliated Hospital (Approval No. wyfy‐2022‐ky‐260), in compliance with the Declaration of Helsinki and relevant national regulations.

## Consent

Written informed consent was obtained from all participants prior to their inclusion in the study. Participants were fully informed of the research purpose, procedures, potential risks, and benefits, and were assured of confidentiality and voluntary participation.

## Conflicts of Interest

The authors declare no conflicts of interest.

## Data Availability

The datasets generated and/or analyzed during the current study are not publicly available due to the following restrictions: (1) Ethical compliance: This study involves sensitive medical records of pregnant women and neonates, which contain personally identifiable information protected under China's Regulations on Human Genetic Resources Management and the Ethical Review Measures for Life Sciences and Medical Research Involving Humans (2023). Full anonymization of these datasets would compromise their scientific validity for longitudinal studies. (2) Institutional Policies: Data sharing is restricted by the Institutional Review Board of Shandong Second Medical University Affiliated Hospital (Approval No. wyfy‐2022‐ky‐260), which mandates custodianship of perinatal health data to prevent unauthorized secondary use. Qualified researchers may request access to de‐identified datasets through the following process: (1) Submit a formal application to the corresponding author (lirx@sdsmu.edu.cn), including: a scientifically valid research proposal; Institutional Ethics Committee. (2) approval certificate; signed Data Use Agreement. The Data Access Committee will conduct a dual review within 30 working days, assessing: (1) Alignment with original study objectives. (2) Data security protocols (e.g., ISO 27001 compliance). (3) Conflicts of interest disclosures.
